# Self-Powered Wearable Biosensor in a Baby Diaper for Monitoring Neonatal Jaundice through a Hydrovoltaic-Biosensing Coupling Effect of ZnO Nanoarray

**DOI:** 10.3390/bios12030164

**Published:** 2022-03-06

**Authors:** Zirui Ning, Zhihe Long, Guangyou Yang, Lili Xing, Xinyu Xue

**Affiliations:** 1 School of Physics, University of Electronic Science and Technology of China, Chengdu 611731, China; 202022120334@std.uestc.edu.cn (Z.N.); yanggy127@163.com (G.Y.); xinglili@uestc.edu.cn (L.X.); 2 Department of Mechanical Engineering, City University of Hong Kong, 83 Tat Chee Avenue, Kowloon, Hong Kong 999077, China; zh.long@my.cityu.edu.hk

**Keywords:** self-powered, neonatal jaundice monitoring, ZnO nanoarray, hydrovoltaic effect, intelligent diaper

## Abstract

Neonatal jaundice refers to the abnormality of bilirubin metabolism for newborns, and wearable transcutaneous bilirubin meters for real-time measuring the bilirubin concentration is an insistent demand for the babies’ parents and doctors. In this paper, a self-powered wearable biosensor in a baby diaper for real-time monitoring neonatal jaundice has been realized by the hydrovoltaic-biosensing coupling effect of ZnO nanoarray. Without external power supply, the system can work independently, and the hydrovoltaic output can be treated as both the power source and biosensing signal. The working mechanism is that the hydrovoltaic output arises from the urine flowing on ZnO nanoarray and the enzymatic reaction on the surface can influence the output. The sensing information can be transmitted through a wireless transmitter, and thus the parents and doctors can treat the neonatal jaundice of babies in time. This work can potentially promote the development of next generation of biosensors and physiological monitoring system, and expand the scope of self-powered technique and smart healthcare area.

## 1. Introduction

Neonatal jaundice refers to the abnormality of bilirubin metabolism (the blood bilirubin level rising) for newborns [1,2,3]. Pathological jaundice can make the face and trunk of the baby appear yellow color, accompanied with anemia, hepatosplenomegaly and yellow urine. In severe cases, it is manifested as poor response, listlessness, anorexia and even breathing difficulties [4,5,6]. Due to unpredictable pathological recurrence in several weeks after birth, it is inconvenient for the hospital to carry out examinations in a timely manner [7,8]. Thus, monitoring and uploading the neonatal jaundice status in real time is an insistent demand for the parents and doctors. At present, the conventional method for jaundice detection is based on blood sampling and bilirubin analysis [9,10]. This technique cannot meet the requirement of real-time dynamic monitoring, and the frequent blood drawing with skin damage is unacceptable for children and their parents. To solve the problem, a new wearable transcutaneous bilirubin meter that can be conformably attached on the skin for real-time measuring the bilirubin concentration in body fluid such as urine is highly expected [11].

The urine bilirubin concentration in the baby with pathological neonatal jaundice obviously rise [12,13], and the diaper is usually worn on a baby for holding urine. Thus, a wearable biosensor embedded in baby diaper for continuously detecting bilirubin concentration can realize monitoring neonatal jaundice in real time. Nowadays, some research groups and companies have developed intelligent diapers with various bilirubin biosensor integration. The main working principal of the biosensor is based on traditional electrochemical or chemical colorimetric approaches [14,15,16,17,18]. These methods provide relatively accurate sensing results, but usually need extra and big-sized bulky power supplies, e.g., a battery or capacitor, which raises the cost of the whole system, enlarges the total volume of the diaper and may also cause harm to the health of baby. Recently reported self-powered techniques may potentially remove the power supply from the system [19,20,21,22,23,24]. The urine bilirubin analyzing sites with wireless, battery-free electronics are promising as substitutions by coupling the energy harvesting and biosensing processes in the diaper environment.

In this paper, a self-powered wearable biosensor in a baby diaper for real-time monitoring neonatal jaundice has been realized by the hydrovoltaic-biosensing coupling effect of ZnO nanoarray. The system can work without an external power unit, and has low cost, small size, noninvasive, and flexible features. The working mechanism is that the urine flowing on ZnO nanoarray and the enzymatic reaction (bilirubin and bilirubin oxidase) on the surface can generate bilirubin dependent hydrovoltaic output [25,26,27,28,29,30,31]. The output can be treated as not only the energy for the sensing process but also the biosensing signal. ZnO nanostructures have the characteristics of hydroelectric effect with high output [32,33]. It also can be easily and efficiently functionalized for sensing by surface chemical modification [34]. The main goal of the device is to monitor the bilirubin in urine to determine whether there is a possibility of jaundice in baby. The signal collected by the sensing unit can be transmitted to the wireless transceiver module, and then transmitted to the guardian’s smart device. Once an abnormality is detected, it can be sent to the doctor for treatment in time. This achievement can play an important role in monitoring neonatal pathological jaundice, helping parents of newborns to obtain information about the baby’s fluid in real time, and sending it to the doctor in the event of an early warning. Meanwhile, this work can expand the scope of self-powered techniques and smart healthcare areas.

## 2. Experimental

### 2.1. Fabrication of Self-Powered Bilirubin Biosensing Unit

Bilirubin oxidase and bilirubin were provided by Chongqing Amida Biotechnology Co., Ltd (Chongqing, China). The other chemicals were provided by Chengdu Keweizhuo Technology Co., Ltd. (Chengdu, China).

The vertically grown ZnO nanoarray was prepared by a hydrothermal method. A piece of PDMS film attached to a silicon wafer (for keeping the film steady in the solution) was cleaned with deionized water and alcohol, and dried at 60 °C. 0.5 g of Zn(NO_3_)_2_·6H_2_O was dissolved in 38 mL of deionized water, then 2 mL of NH_3_·H_2_O was dropped into the solution while stirring. After Zn(NO_3_)_2_·6H_2_O was evenly dissolved, the PDMS film was immersed in the solution. The beaker was quickly sealed and placed in a dry oven, and then kept at 80 °C for 24 h. The PDMS film with ZnO nanoarray grown was finally taken out from the beaker, and stripped off from the silicon wafer [25,35]. 

The ZnO-grown PDMS film was cut into 20 mm × 30 mm in area, and then ZnO nanoarray were modified with bilirubin oxidase (BOx). Lyophilized bilirubin oxidase powder was dissolved in PBS buffer to form 40 u/mL BOx solution. 0.5 mL BOx solution was evenly and slowly dropped on the nanowires. The film was naturally dried for 3–4 h, and the enzyme was modified on the nanowires [36]. Both ZnO and PDMS have been proven to be nontoxic/biocompatible and can work well on the human body environment [37,38,39].

### 2.2. Characterization and Measurements

The morphology and microstructure of ZnO nanoarray were investigated by a scanning electron microscope (Gemini SEM300, Oberkochen, Germany). The output hydrovoltaic voltage is measured with electrometer (Keithley 6514, Beaverton, OR, USA).

## 3. Results and Discussion

### 3.1. Experimental Design

**Figure 1:**
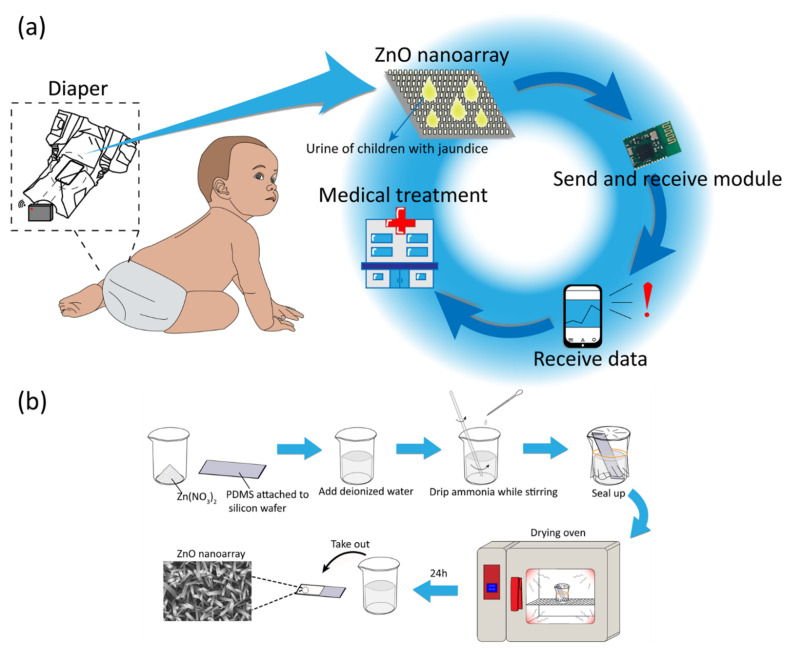
(**a**) The experimental design of self-powered wearable biosensor in baby diaper for monitoring neonatal jaundice. (**b**) Manufacturing process of ZnO nanoarray on PDMS film.

Figure 1a shows the experimental design and application of self-powered wearable biosensor in baby diaper for real-time monitoring neonatal jaundice. The biosensing unit is embedded in a diaper, and urine can flow across the surface of the device. The output hydrovoltaic voltage of the device can be influenced by the concentration of the target biomolecule (bilirubin) in the urine, serving as the biosensing signal. Biological sensor information can be wirelessly transmitted to parents for determining the health of their baby, realizing immediate treatment in time.

Figure 1b shows the typical manufacturing process of the biosensing unit. The vertically grown ZnO nanoarray on PDMS substrate are prepared by a hydrothermal method. Then ZnO nanoarray are modified with BOx by dropping BOx/PBS solution on the nanowires. The detailed process can be found in the experimental section [25,35,36].

### 3.2. Device and Material

**Figure 2:**
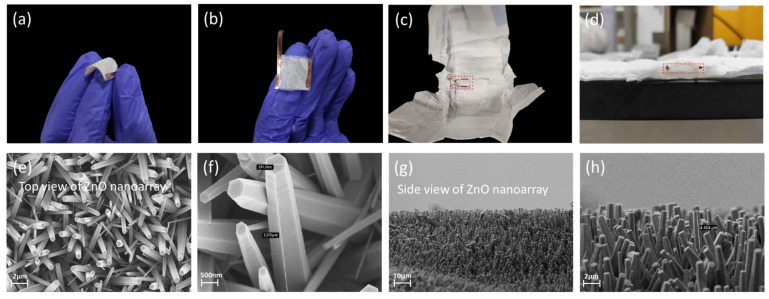
Morphology and microstructure of the self-powered wearable biosensor in baby diaper. (**a**) Side view of the flexible device. (**b**) Top view of the device. (**c**,**d**) The device embedded in baby diaper. (**e**–**h**) SEM images of ZnO nanoarray.

Figure 2 shows the morphology and microstructure of the self-powered wearable biosensor in the baby diaper. Figure 2a,b show that the device has good flexibility and small size. The device can be embedded in the absorbent layer of baby diaper, and can fit well with the diaper, as shown in Figure 2c,d. The top and side views of ZnO nanoarray are shown in the SEM image of Figure 2e–h. The length of the nanowires is about 3 μm, and the cross section of the nanowires is of hexagonal structure with an average diameter of about 600 nm. The area of the ZnO nanoarray is determined by the size of the substrate. In practical applications, the output mainly depends on the area of the liquid flowing. The thickness of ZnO nanoarray is determined by preparation process. The ZnO nanoarray on the device is densely distributed, which is beneficial for the piezoelectric output.

### 3.3. Working Mechanism

**Figure 3:**
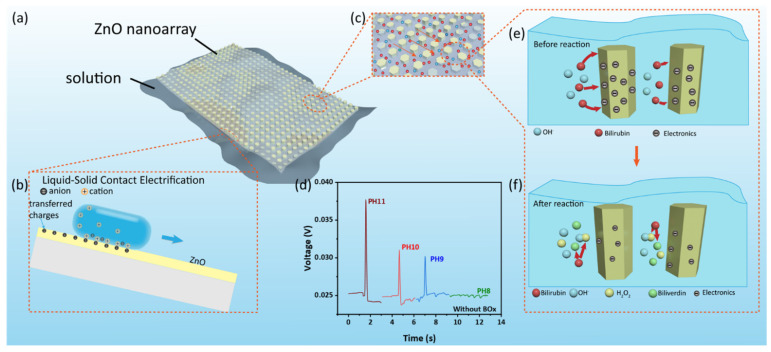
The working mechanism of the self-powered wearable biosensor. (**a**,**b**) The power generating process. (**c**) Molecular flowing along ZnO nanowire. (**d**) The output voltage of the device in different pH value aqueous solution. (**e**,**f**) The biosensing process for detecting bilirubin.

Figure 3a,b show the power generating process of the self-powered wearable biosensor. When the liquid is drawn across the surface of ZnO nanoarray, due to the CE (contact electrification) effect, the free electrons in ZnO can move to the vicinity of the contact region between the droplet and ZnO, resulting in charge transfer. Studies have shown that materials with free electrons will be negatively charged due to the solid–liquid contact-electrification on the surface of various materials [40,41,42,43,44]. In the solid–liquid contact between ionic solution and solid has both ion transfer and electron transfer, while the charge transfer between nonionic liquid and solid is substantially contributed to by electron transfer. A slight increase in ion concentration can increase the amount of CE charge. Furthermore, a large amount of excessive ion concentration in the solution can cause electrons to combine with ions, forming a shielding effect, hindering the charge transfer and inhibiting the amount of CE charge [45,46,47,48]. On the surface of ZnO, the transfer of hydrogen ions and electrons is a competitive process, and hydrogen ions inhibit the transfer of electrons between ZnO and water molecules. In contrast, hydroxide ions do not inhibit this process, but promote this process at high concentration [49]. In order to prove this speculation, we carry out subsequent experiments related to hydroxide ions on the surface of ZnO nanoarray.

Figure 3c–f shows the biosensing process of the device. When the device is in contact with sodium bilirubin, an enzymatic reaction between BOx and bilirubin will occur. The reaction is as follows [29]:

(1) $$Bilirubin+O_{2}\;\overset{BO_{x}}{\to }\;Biliverdin+H_{2}O_{2}$$

Furthermore, hydrogen peroxide is oxidized as follows:

(2) $$H_{2}O_{2}\;\to \;O_{2}+2H^{+}+2e^{-}$$

The hydrogen ion neutralizes the hydroxide ion in the solution thereby affecting the output voltage of the contact electrification. Figure 3d experimentally confirms that hydroxide ions can indeed affect the output voltage of the device. As the pH value of aqueous solution is 11, 10, 9 and 8, respectively, the change of the output voltage is 0.013, 0.006, 0.005 and 0.001 V. These results prove that the biosensing behavior of the device can be attributed to the coupling of enzymatic reaction and hydrovoltaic effect.

### 3.4. Sensing Performance

**Figure 4:**
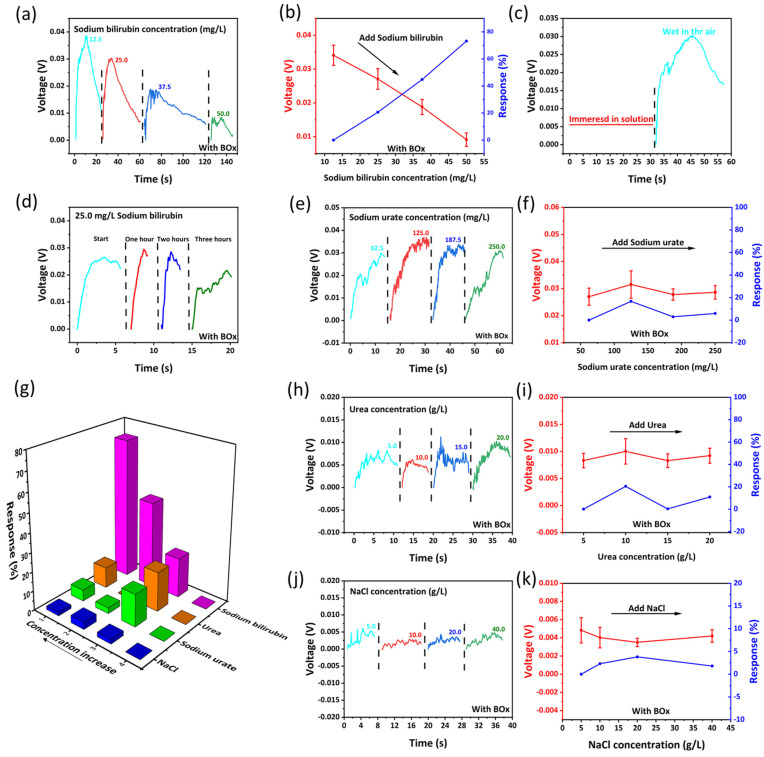
(**a**) The sodium bilirubin biosensing performance of the self-powered wearable biosensor. (**b**) The response of the device. (**c**) Control experiment of the device eliminate primary cell effect. (**d**) The repeatability of the device. (**e**–**k**) The response of the device against sodium urate, urea and NaCl.

Figure 4 shows the biosensing behavior of the self-powered wearable biosensor in baby diaper. Figure 4a shows the biosensing performance of the device for detecting bilirubin (ZnO nanoarray are modified with bilirubin oxidase). Since bilirubin is insoluble in water, NaOH solution (0.004 mol/L) is usually used to dissolve bilirubin in deionized water [50]. The obtained sodium bilirubin is conjugated bilirubin in the solution for the following tests. As shown in Figure 4a, when the concentration of sodium bilirubin dropped on the surface of the device is 12.5, 25.0, 37.5 and 50.0 mg/L, the output hydrovoltaic voltage (peak value) of the device through contact electrification CE is 0.039, 0.030, 0.019 and 0.008 V, respectively. The output voltage is negatively correlated with the concentration of sodium bilirubin in the solution, as shown in Figure 4b. There is an approximately linear relationship between the output voltage and the concentration of sodium bilirubin. The biosensing response can be simply defined as [51]:

(3) $$R\%=\left|\frac{V_{i}-V_{1}}{V_{1}}\right|\times 100\%$$

In Equation (3), V_i_ and V_1_ are the output voltage of the device in each concentration and the initial concentration, respectively. As the output voltage is 0.03408, 0.02708, 0.01881 and 0.00914 V (the average peak value of 10 experiments for each concentration), the response is 0.0%, 20.6%, 44.8%, 73.2%, respectively. By fitting the response scatter plot, the linear regression equation can be obtained as y = 0.2438x − 0.263 [52]. In order to eliminate the influence of the primary cell effect, the device is, respectively, immersed in the solution and taken out (the device is wet in air atmosphere). The output voltage of the device is shown in Figure 4c. It can be seen that the output voltage of the device in the solution is much smaller than that in air. This result confirms that the output voltage is mainly dominated by the contact electrification (CE) effect between the solution and nanowires (a kind of hydrovoltaic effect), and the influence of the primary cell effect can be ignored. Figure 4d shows the repeatability of the device. The device is investigated with same concentration of sodium bilirubin solution for four times (one-hour interval for each test). It can be seen that the device can maintain a stable output within a certain period of time. The repetition number of the device can match the utility of the diaper, and it can be thrown away with the diaper.

Specificity is also one of the important indicators of biosensors. The output voltage of our device is only related to the concentration of sodium bilirubin in the solution, and the device can specifically target bilirubin in solution without being affected by other partial substances. Figure 4e–k shows the specificity of the device against sodium bilirubin. The specificity arises from the enzymatic reaction between bilirubin and bilirubin oxidase. Several other typical substances in urine are dropped on the device at different concentrations, and the output voltage almost keeps unchanged. Figure 4e,f show that as the sodium urate concentration is 62.5, 125.0, 187.5 and 250.0 mg/L, the output voltage (peak value) of the device is 0.0270, 0.0315, 0.0278 and 0.0286 V, and the response is 0.0%, 16.7%, 2.9%, 5.9%, respectively. In Figure 4h,j, as the urea concentration in solution is 5.0, 10.0, 15.0 and 20.0 g/L, the output voltage of the device is 0.0083, 0.0100, 0.0083 and 0.0092 V, and the response is 0.0%, 20.4%, 0.2%, 10.8%, respectively. Figure 4j,k show that as the NaCl concentration in solution is 5.0, 10.0, 20.0 and 40.0 g/L, the output voltage of the device is 0.0048, 0.0040, 0.0035 and 0.0042 V, and the response is 0.0%, 2.3%, 3.8%, 1.8%, respectively. The above peak values are all taken from the average value of five experiments at the corresponding concentrations. The results confirm that the output voltage can be slightly influenced by the other substances in urine. 

**Figure 5:**
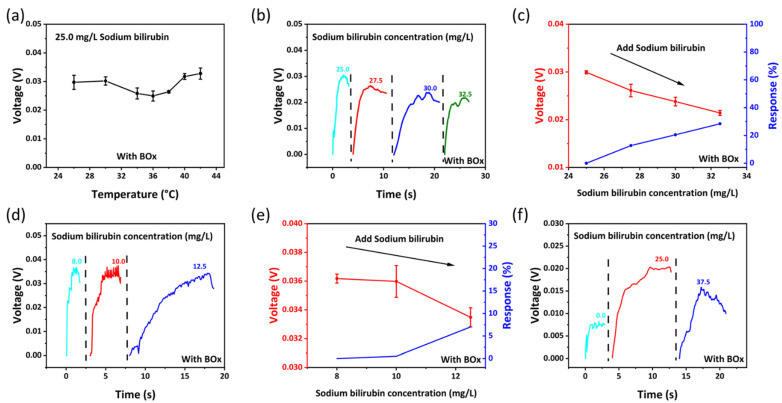
(**a**) The output of the device at different temperatures. (**b**,**c**) The biosensing behavior and response of the device against small lactate concentration change. (**d**,**e**) The limit of detection of the device. (**f**) The biosensing behavior of the device in mixed solution (approximately urine).

Figure 5a shows the output voltage of the device at different temperature. The temperature has a slight influence on the output of the device. Figure 5b,c show the biosensing behavior of the device against small lactate concentration change. As the sodium bilirubin concentration in solution is 25.0, 27.5, 30.0 and 32.5 mg/L, the output voltage of the device is 0.0299, 0.0261, 0.0238 and 0.0214 V, and the response is 0.0%, 12.7%, 20.5%, 28.4%, respectively. Figure 5d,e show the limit of detection of the device, as the sodium bilirubin concentration in solution is 8.0, 10.0 and 12.5 mg/L, the output voltage of the device is 0.0361, 0.0359 and 0.0334 V, the response is 0.0%, 0.5%, 7.0%, respectively. The lower limit of detection concentration of the device is around 8.0 mg/L. Figure 5f shows that when the approximate urine (97% water, 1.8% urea, 0.05% uric acid, 1.1% inorganic salts and trace amounts of sodium bilirubin at the concentrations indicated) containing different concentrations of sodium bilirubin is dropped on the device, the output of the device changes significantly.

### 3.5. Practical Application

**Figure 6:**
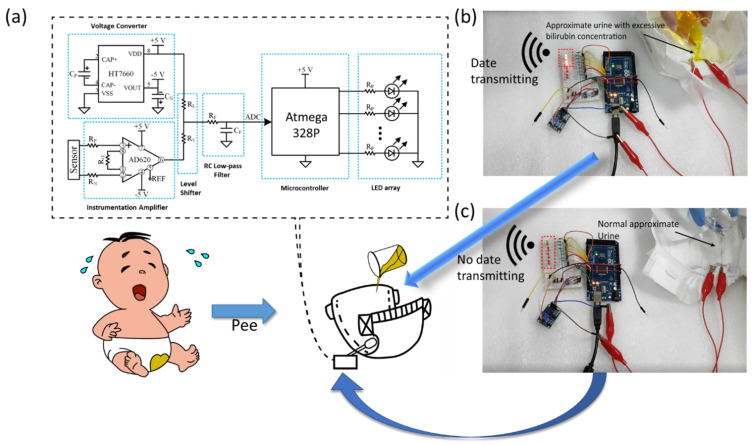
(**a**) Circuit diagram for sensing information transmission. (**b**,**c**) After dropping sodium bilirubin solution on the diaper, the concentration can be read out from the number of LED lights.

Figure 6 shows the practical application of the self-powered wearable biosensor in baby diaper for real-time monitoring bilirubin concentration. The device is embedded between the outer cotton cloth and the inner absorbent layer of the diaper. The device must be placed on top of the absorbent layer, otherwise no liquid can flow on the surface of the device. For uploading the biosensing information, the device is connected to an external circuit module, as shown in Figure 6a. The circuit can amplify the sensing signal, and the single-chip microcomputer can perform analog-to-digital conversion and analysis of the signal through voltage converter, shifter and low-pass filter. Then the circuit can control a wireless transmitter for uploading the sensing information. Here, LED lights are used to exhibit the sensing result. As the single-chip microcomputer detects the amplified signal, LED lights can be lightened on. In our experiment, eight LED lights are lined up. The green light indicates that a voltage with a large variation range is detected (>15 mV). Furthermore, the number of red light represents the output voltage (one red light for 5 mV, two red lights for 10 mV, three red lights for 15 mV and so on). As shown in Figure 6b, after dropping sodium bilirubin solution on the diaper, the concentration can be read out from the number of LED lights. As the solution is prepared to simulate urine containing 1.0 mg/L of sodium bilirubin, three red lights are on. In Figure 6c, as the sodium bilirubin concentration in the urine is zero, no red lights are on. These data demonstrate that this system can roughly analyze the bilirubin concentration in urine. In the near future, other wireless transmitters, such as a Bluetooth module, can be integrated into the system. These techniques can transmit sensing information (bilirubin in urine) to the smart phones of babies’ parents. The parents can real-time monitor the baby’s health and will not delay the treatment of neonatal jaundice.

## 4. Conclusions

In summary, we report a self-powered wearable biosensor in a baby diaper for real-time monitoring neonatal jaundice. The working mechanism is based on the hydrovoltaic-biosensing coupling effect of ZnO nanoarray. The system can work without an external power supply, and the hydrovoltaic output can be treated as the biosensing signal. The biosensor in the diaper can continuously detect bilirubin concentration in urine, and the sensing information can be wirelessly uploaded, which facilitates the parents and doctors treating the neonatal jaundice of baby in time. This self-powered biosensing system can probably expand the scope of intelligent health care.

## Data Availability

The experimental data is contained within the article.
